# Supplementary material for: Veterinary Clinics as Reservoirs for Pseudomonas aeruginosa: A Neglected Pathway in One Health Surveillance
Source: Antibiotics (Basel). 2025 Jul 17;14(7):720. doi: 10.3390/antibiotics14070720 (PMC12291923; doi:10.3390/antibiotics14070720)
Supplement: Supplementary file 1 [file antibiotics-14-00720-s001.zip › SF1. Data aquisition perstistance timeline.pdf]

Data acquisition and processing

Values derive from primary data collected at the Cluj-Napoca (Romania) Veterinary Teaching Hospital (1 June–17 August 2024) (unpublished data).

Weekly surveillance cultures were obtained from ten fixed environmental sites (five ICU sink drains and five operating-room floor zones). At each site a 25 cm^2 area was sampled with a pre-moistened cellulose sponge, eluted into 10 mL neutralising broth, and cultured on cetrimide agar at 37 °C for 48 h. *Pseudomonas aeruginosa* colonies were screened for multidrug resistance (MDR) by disk diffusion (EUCAST v13.0) and confirmed as belonging to the outbreak clone by multilocus sequence typing.

Graphic generation

The heat-map was rendered with **Matplotlib 3.9.0** using a binary, colour-blind-safe palette (white = no detection; dark purple #4A1486 = detection). Cell borders (0.5 pt light-grey) aid readability. The figure canvas was 12 × 6 in with 300 ppi export for on-screen versions and 600 ppi (PNG) for print layouts.

Interpretation

Rows correspond to individual sampling points, columns to sequential surveillance weeks. Dark cells denote at least one recovery of the MDR clone from that site in a given week, revealing sustained colonisation of ICU sinks and sporadic contamination of operating-room floors.
