# Supplementary material for: Veterinary Clinics as Reservoirs for Pseudomonas aeruginosa: A Neglected Pathway in One Health Surveillance
Source: Antibiotics (Basel). 2025 Jul 17;14(7):720. doi: 10.3390/antibiotics14070720 (PMC12291923; doi:10.3390/antibiotics14070720)
Supplement: Supplementary file 1 [file antibiotics-14-00720-s001.zip › SF2. Multi-panel interpretation.pdf]

Figure 2. Multi-panel overview of nosocomial *Pseudomonas aeruginosa* in veterinary settings – detailed explanation

Panel A: Anatomy of a surgical-site infection

This schematic depicts how a postoperative incision can become seeded by environmental P. aeruginosa through two primary routes. The gray rectangle represents the operating-room (OR) table where surgery occurs, with a green "Recovery Mat" positioned immediately below. An aquamarine "Sink" icon on the back wall indicates a high-touch, moisture-rich zone prone to harboring biofilms. A pink circle labeled "Jar" denotes an ointment container placed on a countertop near the surgical field.

A red arrow labeled "Splash contamination" traces the path from the sink/drain area toward the OR table, illustrating how microscopic droplets or aerosols carrying bacteria can travel from the sink to the patient's incision or recovery surface.

An orange arrow labeled "Ointment contamination" shows how opening and handling a topical medication jar on a contaminated countertop can transfer P. aeruginosa directly into an animal's ear canal or onto a fresh wound.

Together, these elements highlight that even minimal contact between a sterile surgical site and adjacent contaminated surfaces (sink splashes or ointment jars) can introduce biofilm-competent bacteria into an incision, potentiating surgical-site infections.

Panel B: Device-mediated transmission in the ICU

This diagram illustrates transmission pathways of P. aeruginosa within a veterinary intensive-care unit (ICU). Three adjacent cages represent critical-care kennels.

For Cage 1, a red line depicts a ventilator circuit where condensate can accumulate (highlighted by a blue "Water Trap"). A blue arrow labeled "Condensate" points from the water trap to Cage 1, showing how moisture in the ventilator tubing can harbor and deliver bacteria into a patient's airway when aerosolized.

For Cage 3, a yellow "Catheter" icon inside the kennel represents an indwelling intravenous line. A purple arrow labeled "Sink Counter contamination" leads from a not-shown sink counter toward the catheter, indicating that clinicians placing the catheter flush syringe on a contaminated counter may introduce P. aeruginosa into the catheter lumen.

This panel highlights how shared equipment (ventilators, humidifier water traps) and momentary lapses (placing syringes on contaminated surfaces without glove changes) can serve as hidden transmission nodes in high-risk ICU environments.

Panel C: Documented Outbreak Case Summaries

This timeline visualizes three real-world outbreak tracks (Endophthalmitis, Ventilator-Associated Pneumonia [VAP], and Pool Contamination) over 12 weeks. The horizontal axis represents weeks (0–12), while each colored marker (blue, orange, green) corresponds to a specific cluster of cases:

Endophthalmitis (blue, Track 1 at y = 1)

Week 0.5: "Bottle Purchased" marks the acquisition of a non-sterile ophthalmic irrigation solution.

Week 2: "1st Case" denotes the first canine postoperative endophthalmitis.

Week 3: "Outbreak ID" indicates whole-genome sequencing confirmation linking infections to the same source.

Ventilator-Associated Pneumonia (orange, Track 2 at y = 2)

Week 1: “Cracked Elbow” identifies discovery of a fracture in the humidifier elbow within the ventilator circuit.

Week 2: “1st VAP” marks the first dog diagnosed with ventilator-associated pneumonia.

Week 3.5: “Circuit Replaced” shows the corrective action taken (full circuit replacement and use of heat-moisture exchanger filters).

Pool Contamination (green, Track 3 at y = 3)

Week 0: “Routine Use” indicates standard operation of a hydrotherapy pool without incident.

Week 4: “2 Otitis” marks two dogs developing persistent otitis associated with pool biofilm contamination.

Week 5: “Pool Cleaned” denotes draining, high-pressure steam cleaning, and reopening of the pool.

Dotted vertical grid lines help align events to specific weeks. Each track’s markers and labels demonstrate how environmental reservoirs (ophthalmic solution, cracked humidifier elbow, hydrotherapy pool) served as outbreak nuclei, and how targeted interventions (discarding contaminated solution, replacing equipment, deep cleaning) led to resolution of each cluster.
