# Supplementary material for: Veterinary Clinics as Reservoirs for Pseudomonas aeruginosa: A Neglected Pathway in One Health Surveillance
Source: Antibiotics (Basel). 2025 Jul 17;14(7):720. doi: 10.3390/antibiotics14070720 (PMC12291923; doi:10.3390/antibiotics14070720)
Supplement: Supplementary file 1 [file antibiotics-14-00720-s001.zip › SF3. Conceptual diagram of One Health transmission pathways for MDR Pseudomonas aeruginosa.pdf]

Figure 3. Conceptual diagram of One Health transmission pathways for MDR *Pseudomonas aeruginosa*

This infographic illustrates the interconnected roles of **animals**, **humans**, and the **environment** in the spread of multidrug-resistant (*MDR*) *Pseudomonas aeruginosa* within a One Health framework.

- **Center Node — Veterinary Clinic**: Positioned as the central hub, the clinic receives and outputs *P. aeruginosa* strains via multiple vectors. Infected or colonized animals enter with environmental or household strains; these organisms can persist and amplify on surfaces (e.g., drains, exam tables), be spread through contaminated tools or clothing, and exit with patients, staff, or waste.
- **Animal Pathway**: Companion animals, shelter populations, and wildlife patients can introduce or acquire *P. aeruginosa* strains through wounds, otitis, surgical sites, or raw diets. Once colonized, they may carry these strains back into homes, foster systems, or the broader urban ecosystem.
- **Human Pathway**: Veterinary personnel and pet owners are exposed through direct contact, aerosolized droplets (from ear flushes or hydrotherapy), or contaminated surfaces. Immunocompromised individuals face elevated risk of invasive infection. Bidirectional transmission is possible when humans work in both veterinary and human healthcare settings or bring contaminated materials home.
- **Environmental Pathway**: Biofilm-forming *P. aeruginosa* can persist in drains, on equipment, and within HVAC or sewer systems. Wastewater from clinics — including laundering runoff or drain effluent — can carry resistant genes into municipal treatment networks and natural water bodies. Shared or adjacent plumbing with human healthcare sites may enable cross-sectoral spread.
- **Pharmaceutical and Infrastructure Nodes**: Shared supply chains (e.g., for compounded medications or disinfectants), teaching facilities, or collaborative research spaces create additional bridges between sectors. A contaminated otic solution or sink line can serve as a common point source across species and institutions.

This visualization reinforces the need for coordinated surveillance and intervention strategies across veterinary, public health, and environmental domains — a foundational principle of the One Health model.
